# Effect of Co Substitution and Thermo-Magnetic Treatment on the Structure and Induced Magnetic Anisotropy of Fe_84.5__−x_Co_x_Nb_5_B_8.5_P_2_ Nanocrystalline Alloys

**DOI:** 10.3390/ma14123433

**Published:** 2021-06-21

**Authors:** Aleksandra Kolano-Burian, Przemyslaw Zackiewicz, Agnieszka Grabias, Anna Wojcik, Wojciech Maziarz, Maciej Szlezynger, Patryk Wlodarczyk, Maciej Kowalczyk, Lukasz Hawelek

**Affiliations:** 1 Lukasiewicz Research Network—Institute of Non-Ferrous Metals, 5 Sowinskiego, 44-100 Gliwice, Poland; przemyslaw.zackiewicz@imn.lukasiewicz.gov.pl (P.Z.); patryk.wlodarczyk@imn.lukasiewicz.gov.pl (P.W.); lukasz.hawelek@imn.lukasiewicz.gov.pl (L.H.); 2 Lukasiewicz Research Network—Institute of Microelectronics and Photonics, al. Lotnikow 32/46, 02-668 Warszawa, Poland; agnieszka.grabias@imif.lukasiewicz.gov.pl; 3 Institute of Metallurgy and Materials Science Polish Academy of Sciences, 25 Reymonta, 30-059 Krakow, Poland; a.wojcik@imim.pl (A.W.); w.maziarz@imim.pl (W.M.); m.szlezynger@imim.pl (M.S.); 4 Faculty of Materials Science and Engineering, Warsaw University of Technology, 141 Woloska, 05-507 Warszawa, Poland; maciej.kowalczyk@pw.edu.pl

**Keywords:** soft magnetic materials, surface crystallization, thermo-magnetic treatment, induced magnetic anisotropy

## Abstract

In the present work, we investigated in detail the thermal/crystallization behavior and magnetic properties of materials with Fe_84.5-x_Co_x_Nb_5_B_8.5_P_2_ (x = 0, 5, 10, 15 and 20 at.%) composition. The amorphous ribbons were manufactured on a semi-industrial scale by the melt-spinning technique. The subsequent nanocrystallization processes were carried out under different conditions (with/without magnetic field). The comprehensive studies have been carried out using differential scanning calorimetry, X-ray diffractometry, transmission electron microscopy, hysteresis loop analyses, vibrating sample magnetometry and Mössbauer spectroscopy. Moreover, the frequency (up to 300 kHz) dependence of power losses and permeability at a magnetic induction up to 0.9 T was investigated. On the basis of some of the results obtained, we calculated the values of the activation energies and the induced magnetic anisotropies. The X-ray diffraction results confirm the surface crystallization effect previously observed for phosphorous-containing alloys. The in situ microscopic observations of crystallization describe this process in detail in accordance with the calorimetry results. Furthermore, the effect of Co content on the phase composition and the influence of annealing in an external magnetic field on magnetic properties, including the orientation of the magnetic spins, have been studied using various magnetic techniques. Finally, nanocrystalline Fe_64.5_Co_20_Nb_5_B_8.5_P_2_ cores were prepared after transverse thermo-magnetic heat treatment and installed in industrially available portable heating equipment.

## 1. Introduction

The global trend of miniaturization and mobility is limited by the development of new efficient materials such as amorphous and nanocrystalline soft magnetic materials. These materials play an increasingly important role in the broadly understood area of energy conversion. However, due to their applicability, the price–performance ratio is usually decisive [1,2,3,4]. One of the most important areas of development is the search for relatively cheap and easy-to-produce materials with the highest possible magnetic saturation. These materials, due to their excellent soft magnetic properties, such as high magnetic induction Bs and low power losses Ps, show the ability to convert the same amount of energy but in a smaller volume, making them an excellent replacement for conventional materials such as silicon steel. Much effort was put into the development of nanocrystalline alloys with large Bs, e.g., Nanoperm (representative Fe_90_Zr_7_B_3_, Bs = 1.63 T) [5], Hitperm (representative (Fe_0.5_Co_0.5_)_88_Zr_7_B_4_Cu_1_, Bs = 1.64 T) [6], Fe-Ni-Cu-Nb-Si-B, Bs = 1.4 T [7,8], Fe-Co-Cu-Nb(Zr)-Si-B, Bs = 1.53–1.74 T [9] and Fe-Si-B-P-(C)-Cu, Bs = 1.79–1.88 T [10,11]. With the exception of power electronic switches, electrical devices almost always contain passive electric components such as inductors and capacitors. Cores operating in power electronic circuits characterized by a broad spectrum of current/voltage harmonics are components that cause power losses that are difficult to eliminate. Due to the fact that all the energy transferred to the load flows through these coils, it is of great importance to reduce their power losses and their size and mass while maintaining a high level of efficiency. Usually, in specific applications, electricity needs to be converted to meet certain requirements. Induction heating is one of the most demanding applications due to the need to process large amounts of energy in a short time, the possibility of temporary overload and, in the case of mobile devices, the demand for high efficiency, weight and size reduction. Typically, silicon steel cores are used in such applications to enable the use of the highest magnetic saturation. However, increasing productivity and reducing size requires the use of more advanced materials, such as the recently studied Fe-Co-Nb-B-P type alloys [12,13,14]. These materials possess high Bs = 1.7 T, satisfactory mechanical strength, relatively low power losses and an appropriate Tc value, which allows for the improvement of their properties by appropriate heat treatment in the presence of an external transverse magnetic field. This new class of P-containing Fe-rich alloys is relatively easily accessible by conventional casting technology under ambient atmosphere, although the composition is close to the glass formation limit. The latter can result in partial crystallization in the as-cast state, typically on the ribbon surfaces.

This study aimed to determine the correlation between the conditions of thermo-magnetic treatment of Fe_84.5-x_Co_x_Nb_5_B_8.5_P_2_ (x = 0, 5, 10, 15 and 20 at.%) nanocrystalline alloys (proper name: Pyroperm) with the induced transverse anisotropy and magnetic parameters such as core losses Ps, coercivity Hc and the magnetic permeability µ from the point of view of their possible applications in the area of induction heating. The nanocrystallization process of amorphous alloys was investigated by differential scanning calorimetry and transmission electron microscopy techniques, including in situ heating TEM experiments. ^57^Fe Mössbauer spectroscopy measurements were performed to observe changes in the magnetic environment of iron, both in the amorphous and crystalline phases, which were caused by modification of the chemical composition (Co content) of the alloys and their thermal treatment with or without the external magnetic field.

## 2. Materials and Methods

**Figure 1:**
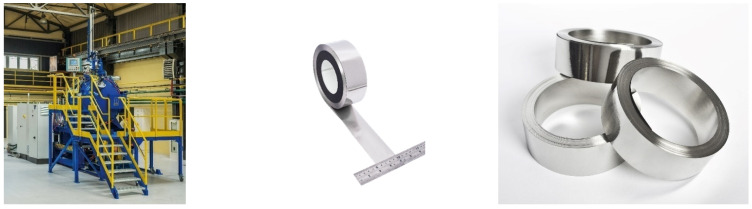
Melt spinning casting setup used in the research, 50.8 mm wide ribbon obtained during the casting, exemplary wound cores prepared for annealing.

The master alloys with nominal composition of Fe_84.5-x_Co_x_Nb_5_B_8.5_P_2_ (x = 0, 5, 10, 15 and 20 at.%) were obtained by melting Fe (3N), Co (3N) and FeB_18_ (2.5N), FeNb_65_ (2.5N), FeP_26_ (2.5N) in the VIM-LAB 50-60 vacuum induction furnace. The as-quenched ribbons with a nominal composition of Fe_84.5__−x_Co_x_Nb_5_B_8.5_P_2_ (x = 0–20 at.%) have been prepared by rapid quenching from the melt on the industrial casting equipment. The ribbons have a width of 50.8 mm and a thickness of 25 µm. Figure 1 shows a melt-spinner with a capacity of 50 kg/cycle and a width of cast strips up to 60 mm, which was designed and built at the Institute of Non-Ferrous Metals (Gliwice, Poland).

For subsequent annealing and testing the dynamic magnetic properties, the as-quenched ribbons were cut into 10 mm wide pieces. Then they were automatically wound into toroidal cores with an inner and outer diameter of 20 mm and 30 mm, respectively. The isothermal annealing of these specimens was carried out in the temperature range from 425 to 725 °C for 20 min in a vacuum furnace (5 × 10^−4^ mbar). The Remacomp C-1200 (MAGNET-PHYSIK Dr. Steingroever GmbH, Köln, Germany) magnetic measurement system was used to determine the magnetic induction Bs, coercivity Hc, remanence Br and power losses Ps. Density values measured for the Fe_84.5__−x_Co_x_Nb_5_B_8.5_P_2_ (x = 0–20 at.%) alloys by the Archimedean method (AccuPyc 1330, Micromeritics) slightly increased from 7.55 g/cm^3^ for the Co-free alloy to 7.75 g/cm^3^ for the highest Co content in the alloy. To investigate the temperature dependence of magnetization, the Curie temperature (Tc) of the sample cut from the optimal core was analyzed with the Physical Property Materials System (PPMS) by Quantum Design, with VSM option. Measurements were made in the heating/cooling mode in the temperature range from 27 to 627 °C. The heating and cooling rate was 5 °C/min. The value of the external field was 50 Oe (4000 A/m). The amorphousness of the as-spun and the structure of annealed ribbons were studied by X-ray diffraction (XRD) at room temperature using a Rigaku MiniFlex 600 diffractometer (Rigaku, Tokyo, Japan) equipped with a copper tube CuKα. The crystallization processes have been monitored by differential scanning calorimetry (DSC) at a heating rate of 5–30 °C/min using the thermal analyzer Netzsch STA F3 Jupiter (NETZSCH-Gerätebau GmbH, Selb, Germany). Transmission electron microscopy (TEM) images in bright field (BF) and dark field (DF) modes and the selected area diffraction patterns (SADPs) were recorded using the Tecnai G2 F20 (200 kV) electron microscope (Thermo Fisher Scientific, Waltham, MA, USA). The in situ experiments were performed using the Gatan 628 heating holder with a heating rate from 20 °C/min to 600 °C. Thin foils for TEM observations were prepared with a TenuPol-5 double jet electropolisher using an electrolyte of perchloric acid (80%) and methanol (20%) at a temperature near −20 °C. Thermo-magnetic annealing was performed on the basis of the thermal conditions for Fe_84.5-x_Co_x_Nb_5_B_8.5_P_2_ (x = 0, 5, 10, 15 and 20 at.%) materials, subjected to heat treatment in a zero magnetic field. Toroidal cores with outer diameter d_o_ = 46 mm and inner diameter d_i_ = 30 were prepared and annealed at the temperature of 525 °C for 20 min. This operation was performed in a protective atmosphere of Ar, in an external magnetic field HT in the range of 94.5–140.3 kA/m. The field was applied along the toroid axis perpendicular to the ribbon casting direction. After such thermo-magnetic treatment, the AC (50 Hz–100 kHz) magnetic properties of the cores were measured using a computerized hysteresis loop tracer (Magnet-Physik’s REMACOMP C-100). The induced magnetic anisotropies Ku were estimated from the measured anisotropy field H_K_ and the saturation magnetization Ms data. The anisotropy field was evaluated from the remanence branch of the hysteresis loop using the singular point method in the mode proposed by Barandiaran et al. [15,16]. Additionally, ^57^Fe Mössbauer spectroscopy was used to compare the phase composition and magnetic properties of the alloys annealed at 525 °C for 20 min without and in the external magnetic field of 125 kA/m. Mössbauer measurements were performed in transmission geometry using a ^57^Co-in-Rh source.

## 3. Results and Discussion

**Figure 2:**
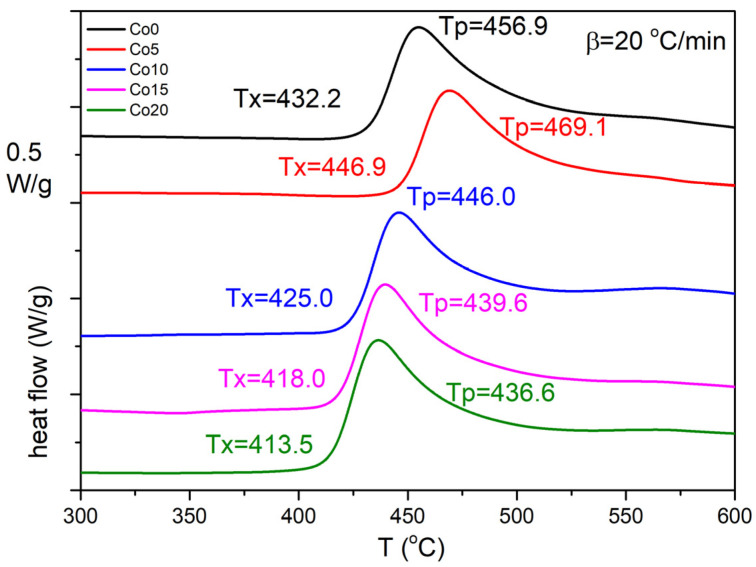
Differential scanning calorimetry curves of Fe_84.5-x_Co_x_Nb_5_B_8.5_P_2_ (x = 0–20 at.%) alloys. The heating rate is 20 °C/min.

The kinetics of non-isothermal crystallization in Pyroperm ribbons with different cobalt content was investigated. In order to analyze the crystallization phenomenon in Pyroperm alloys with different cobalt ratios, differential scanning calorimetry (DSC) measurements have been performed. The measurements of the as-cast ribbons have been performed at different heating rates (5–30 °C/min) in order to evaluate the activation energy of this crystallization. In Figure 2, DSC heating curves with a 20 °C/min heating rate have been shown as an example. The exothermic process, which is clearly seen in Figure 2, has been identified as the nanocrystallization of the α-Fe (for Co0) and α-FeCo (for Co5-Co20) phases. The Kissinger model [17,18] was used to acquire average activation energy, which rises after introducing cobalt to the alloy (Co5) and then drops when the amount of cobalt increases (up to Co20). This method is based on the equation:

(1) $$\ln\left(\frac{\beta }{T_{p}^{2}}\right)=\ln\left(\frac{A_{0}R}{E_{a}}\right)-\frac{E_{a}}{RT_{p}},$$

where: *β* is a heating rate, *T_p_*—temperature of the re-crystallization peak, *E_a_*—activation energy, *R*—gas constant and *A*_0_—pre-exponential factor.

**Figure 3:**
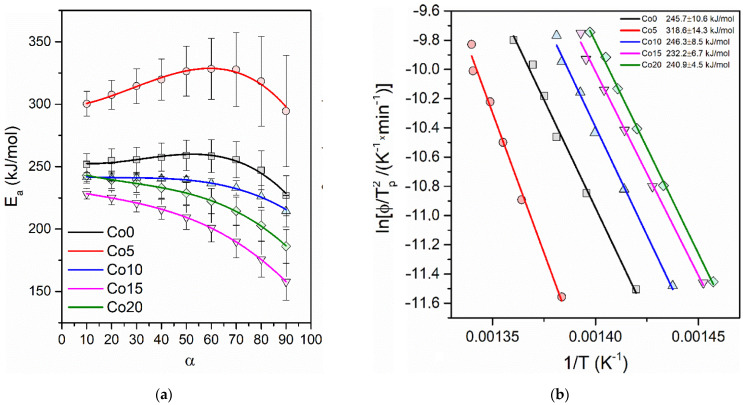
(**a**) The activation energy for the crystallization process derived from the FWO model related to the progress of crystallization; (**b**) The average values of the activation energies derived from the Kissinger model.

Figure 3b shows the average activation energy values derived from the Kissinger model. For more details on this phenomenon, the Flynn–Wall–Ozawa (FWO) model [19,20,21,22] was used to monitor the activation energy changes during crystallization. The FWO model allows for determining activation energy as a function of the degree of crystallinity. Activation energy plots are presented as insets in the FWO fits in Figure 3a. In the case of the Co0 and Co5 alloys, the energy behaves similarly. The activation energy increases during the crystallization process by up to 50% of the crystallization progress. When more cobalt is added to the alloy, there is no longer any characteristic increase in energy during the first stage of crystallization. Moreover, the change in characteristics is related to the decrease of the average value of activation energy. The possible explanation of this phenomenon might be related to the not fully amorphous state of the as-cast ribbons for cobalt-rich alloys. The characteristic increase in energy during the crystallization progress can be related to the nucleation process limiting the rate of crystallization. When nucleation occurs, the activation energy drops drastically. Although the cobalt has to be associated with an increase in the activation energy of the crystallization process, the energy decreases due to a possible partial crystallization, that occurred during the initial quenching of the ribbon.

**Figure 4:**
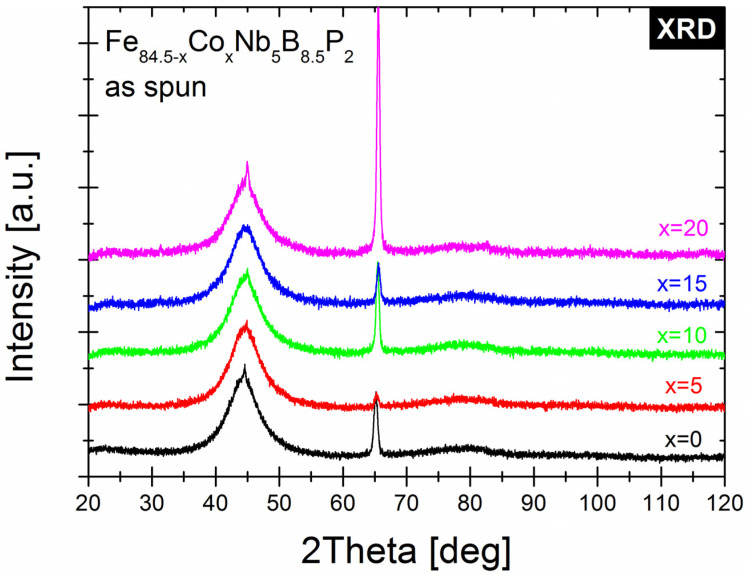
The X-ray diffraction patterns of Fe_84.5__−x_Co_x_Nb_5_B_8.5_P_2_ ribbons in as-spun state.

Figure 4 shows the XRD data for all as-quenched samples. In all cases, the crystalline phase is already present in the as-quenched ribbons, except for the dominant amorphous phase. The surface crystallization of amorphous ribbons has been thoroughly studied for many years, and a number of causes of this phenomenon have been identified [23,24]. In the case of alloys containing phosphorus, a reduction in the content of metalloids in the surface area can be observed due to the selective evaporation of phosphorus during quenching, which is caused by the high vapor pressure of this element. An additional reason for surface crystallization at the “wheel side” of the ribbon was the special cooling of the casting crucible nozzle, with a protective gas eliminating the rapid oxidation of the alloy containing phosphorus.

**Figure 5:**
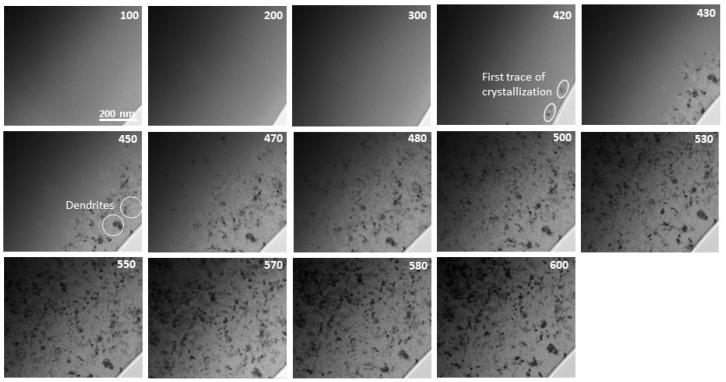
A set of BF images recorded for the Fe_84.5_Nb_5_B_8.5_P_2_ ribbon during in situ heat treatment up to 600 °C.

**Figure 6:**
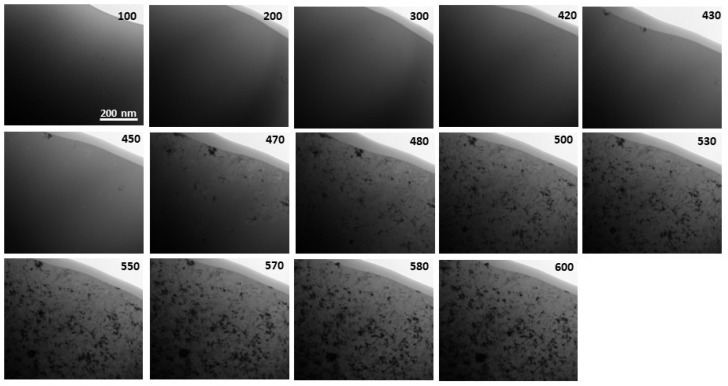
A set of BF images recorded for the Fe_64.5_Co_20_Nb_5_B_8.5_P_2_ ribbon during in situ heat treatment up to 600 °C.

In situ TEM heating experiments at a heating rate of 20 °C/min were performed on Fe_84.5__−x_Co_x_Nb_5_B_8.5_P_2_ (x = 0–20 at.%) as-spun ribbons in the amorphous state. The areas for the observations were carefully selected to avoid the existence of the primary crystallites (observed in Figure 4). Figure 5 and Figure 6 depict the exemplary sets of BF images recorded for Fe_84.5_Nb_5_B_8.5_P_2_ and Fe_64.5_Co_20_Nb_5_B_8.5_P_2_ ribbons during in situ heat treatment up to 600 °C to trace the microstructural evolution during the crystallization process. The crystallization process is the same for all alloys, starting with the appearance of the first crystal nuclei with a size of a few nm. Then, dendritic growth can be observed to reach the fully crystalline state. The onset temperatures of the crystallization process are consistent with the DSC results (see Figure 2). It is worth noting that the crystallization behavior of all studied Fe_84.5__−x_Co_x_Nb_5_B_8.5_P_2_ ribbons was similar regardless of the Co concentration in the alloy.

**Figure 7:**
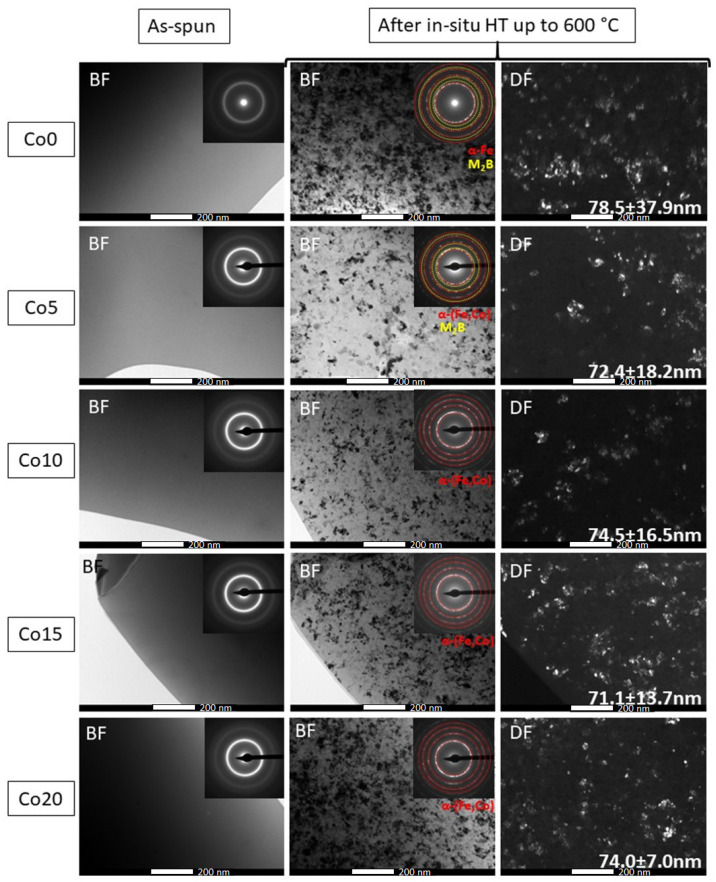
A set of BF, DF and SADP images for as-spun and in situ heat-treated Fe_84.5__−x_Co_x_Nb_5_B_8.5_P_2_ (x = 0, 5, 10, 15, 20 at.%) ribbons.

**Figure 8:**
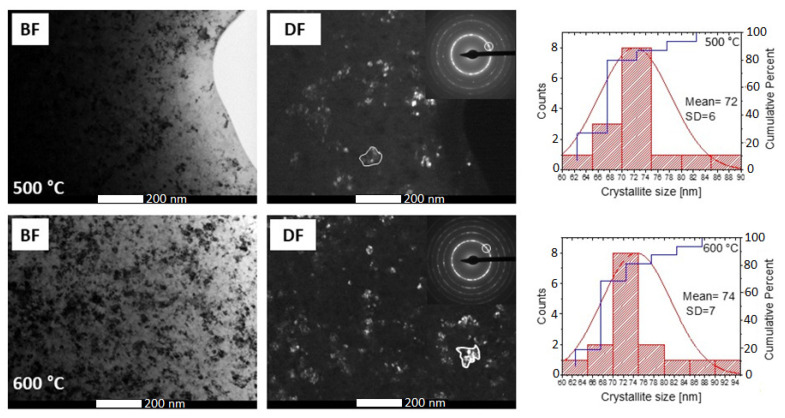
The example of BF and DF images of the Fe_64.5_Co_20_Nb_5_B_8.5_P_2_ ribbon taken at 500 and 600 °C.

**Figure 9:**
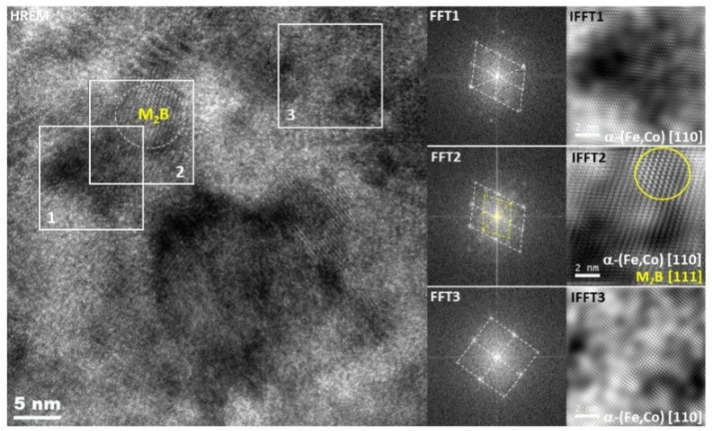
HREM image with corresponding FFT and IFFT taken from marked areas of the Fe_69.5_Co_5_Nb_5_B_8.5_P_2_ ribbon.

Figure 7 presents a set of TEM bright field (BF), dark field (DF) and selected area diffraction pattern (SADP) images of the as-spun and crystallized ribbons after in situ heat treatment up to 600 °C at a heating rate of 20 °C/min. The images corresponding to the as-spun base ribbons show a fully amorphous microstructure with characteristic “halo” rings, while images of the heat-treated alloys reveal completely crystallized microstructures with averaged crystallite sizes of about 78.5 ± 37.9, 72.4 ± 18.2, 74.5 ± 16.5, 71.1 ± 13.7 and 74.0 ± 7.0 nm for ribbons containing 0, 5, 10, 15 and 20 at.% of Co, respectively. Crystallite size was determined from the dark field images. The dendritic shape was confirmed by BF images for selected crystallites. The DF images obtained from a (110)α-(Fe,Co) diffraction ring of the Fe_64.5_Co_20_Nb_5_B_8.5_P_2_ ribbon recorded at 500 and 600 °C are shown in Figure 8. The DF images clearly show contours of the dendritic crystallites, which allowed an objective assessment of the mean crystallite size. From the results, it can be clearly stated that the addition of Co does not significantly affect the size of crystallites. Only minor differences are noticed, which are consistent with the magnetic measurements (see Table 1). Moreover, DF images recorded at two temperatures show that the rapid grain growth occurs up to 500 °C, then the grain size remains almost constant, and only a slight increase of grain size (72 → 74 nm) can be noticed. It can therefore be concluded that the grain size values estimated at 500 °C correspond well to the values measured at 600 °C. In SADP images, the diffraction rings can be indexed according to bcc α-(Fe) and α-(Fe,Co) phases for ribbons with 0 and 5–20 at.% of Co, respectively. However, for the ribbons with 0 and 5 at.% of Co, additional diffraction spots (marked with yellow circles) were detected, which correspond to the M_2_B phase, where M is Fe, Co. The existence of this additional phase has been confirmed based on high-resolution HREM images (Figure 9). Fast Fourier transform (FFT) and inverse fast Fourier transform (IFFT) were generated from the areas marked with squares. It was observed that both the α-(Fe,Co) and (Fe,Co)_2_B phases were detected and identified based on the indexation of IFFT images. The M_2_B phase with the size of 5–15 nm coexists with α-(Fe,Co) crystallites. However, it should be noted that the trace amount of borides is most probably related to local overheating during the in situ process.

**Figure 10:**
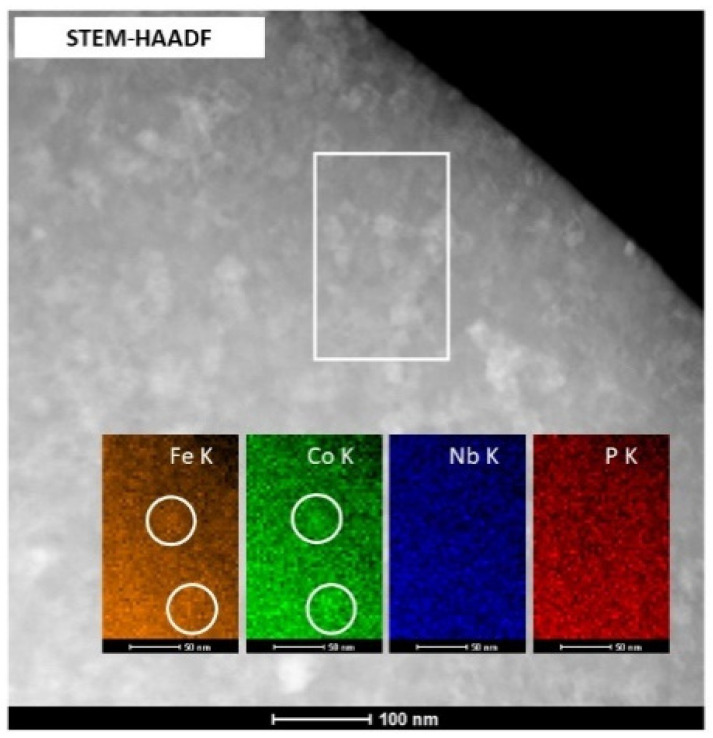
STEM-HAADF image with elemental mapping of Fe_69.5_Co_5_Nb_5_B_8.5_P_2_.

Additionally, the chemical analysis was performed using the EDS/STEM method. Figure 10 shows an example of a STEM-HAADF image for a Fe_69.5_Co_5_Nb_5_B_8.5_P_2_ ribbon with element mapping results, showing that the crystallites are enriched in Fe and Co. At the same time, Nb and P are uniformly distributed in the sample. A boron analysis was not performed here due to a detection limit of the EDS method.

**Figure 11:**
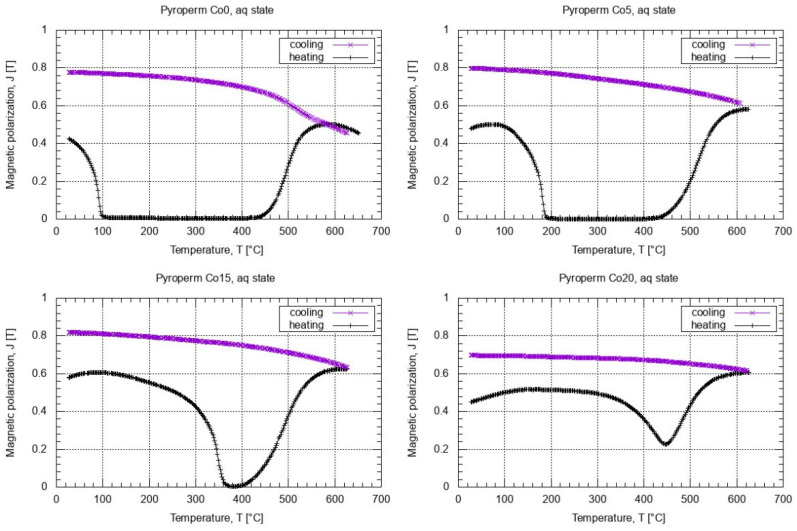
M vs. T curves of Fe_84.5__−x_Co_x_Nb_5_B_8.5_P_2_ (x = 0–20 at.%).

The onset of crystallization can also be followed by studying the thermo-magnetic properties of Co0-Co20 alloys. The as-quenched samples were characterized by PPMS, measuring their magnetization at 50 Oe from room temperature up to 627 °C and then back to room temperature. The M vs. T curves are shown in Figure 11 for all alloys. The ferromagnetic-paramagnetic transition temperature of the alloy increases by approx. 100 °C for every 5% of Co. Due to the fact that the as-spun ribbons are not fully amorphous, as indicated by the XRD results (see Figure 4), the Curie temperature may be higher than the temperature of the first stage of crystallization, which is particularly evident for the Co15 and Co20 alloys. The annealing conditions were optimized on the basis of the results obtained from the studies of crystallization kinetics and thermo-magnetic measurements of Fe_84.5__−x_Co_x_Nb_5_B_8.5_P_2_ series of alloys. Toroidal cores were annealed at various temperatures in the range 425–725 °C for 20 min in a vacuum furnace (5 × 10^−4^ mbar). For all alloys, the a characteristic minimum of Hc was observed, which occurred around the temperature of 455 °C for x = 0–20 at.%. For such annealed samples, the X-ray diffraction measurements proved the presence of α-Fe nanocrystals and the dominant amount of amorphous matrix rich in boron, which is consistent with the in situ TEM observation results (Figure 5 and Figure 6). Table 2 shows the results of the influence of the annealing temperature Ta on the magnetic properties of the Co20 alloy, for which the hysteresis loops have been measured at 50 Hz, with the maximum applied magnetic field of 1500 A/m. In the case of annealing at a temperature that is close to the second crystallization stage onset temperature (Ta = 625 °C), a drastic increase in the coercive field up to 148 A/m was observed, which indicates that in the case of the Co20 alloy the precipitation increases and the alloy becomes magnetically harder. It is also worth mentioning that for a relatively wide Ta range (up to 575 °C) the low Hc value corresponds to the grain size (up to 72–74 nm) observed in the DF images recorded during the in situ TEM experiment. Table 1 compares the effect of the Co content on the magnetic properties of Fe_84.5__−x_Co_x_Nb_5_B_8.5_P_2_ alloys annealed at 455 °C for 20 min.

The evolution of the magnetic properties, especially the coercivity as a function of the annealing temperature depending on the Co content in the alloy was investigated by Kolano-Burian et al. for Fe_73.5__−x_Co_x_Cu_1_Nb_3_Si_13.5_B_9_ and for Finemet-type alloys with x = 0–73.5 at.% [25]. The characteristic minimum of Hc, observed for all studied alloys, corresponded to the optimal temperature of annealing T_a-opt_. For the Fe_84.5__−x_Co_x_Nb_5_B_8.5_P_2_ series of alloys, a similar behavior was observed, as in the case of Fe_73.5__−x_Co_x_Cu_1_Nb_3_Si_13.5_B_9_, for x = 30–58.8 at.%, for which the magnetocrystalline anisotropy plays a major role in the increase of the coercive field. This is because the grain size is almost constant at approx. 8–9 nm for Co-modified Finemet alloys and approx. 70 nm for Pyroperm alloys, and the saturation magnetostriction gradually decreases with increasing Co content in the alloys. For higher Co content in the Fe_84.5__−x_Co_x_Nb_5_B_8.5_P_2_ alloys (x = 40 and 60 at.%), the decrease in the value of the coercive field in relation to the value determined for the alloy with 20 at.% of Co is also a result of lower magnetocrystalline anisotropy [12].

**Figure 12:**
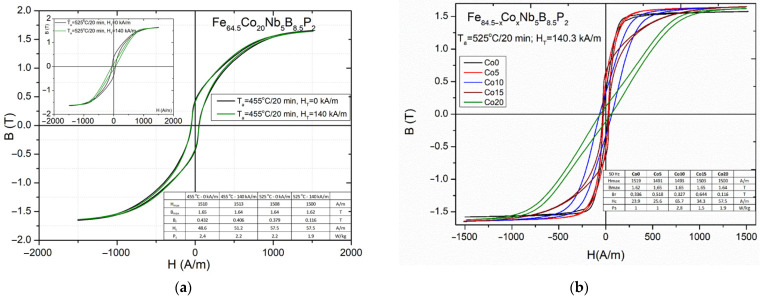
(**a**) Hysteresis loops for a Fe_64.5_Co_20_Nb_5_B_8.5_P_2_ core after annealing at 455 °C without magnetic field (black) and in presence of a transverse magnetic field 140 kA/m (green) and on insert after annealing at 525 °C without magnetic field (black) and in presence of a transverse magnetic field 140 kA/m (green); (**b**) Hysteresis loops for Fe_84.5__−x_Co_x_Nb_5_B_8.5_P_2_ (*x* = 0–20 at.%) cores annealed at 525 °C in a presence of transverse magnetic field 140 kA/m.

From the application point of view, the investigated Fe_84.5__−x_Co_x_Nb_5_B_8.5_P_2_ nanocrystalline cores must have a high saturation induction value of 1.7 T and a flat hysteresis loop to ensure the ability to work under temporary overload conditions. The maximum power reached by the device for a limited time exceeds the continuous output power. In order to obtain flat hysteresis loops characterized by effective permeability even below 10,000, it was necessary to carry out the annealing process in the presence of a transverse magnetic field. Figure 12a presents the effect of the presence of a magnetic field during the thermal treatment of Co20 cores at the optimal temperature, determined to minimize power losses. The presence of a transverse magnetic field during processing under optimal thermal conditions does not affect the shape of the hysteresis loop. The inset in Figure 12a shows the situation obtained after increasing the annealing temperature by 70 °C. The main parameters of hysteresis loops obtained at 455 °C and 525 °C are summarized in the table. For the core processed at the temperature of 525 °C, in the presence of a magnetic field, the Br was obtained at the level of 65–75%, lower than in the other samples. Figure 12b presents the hysteresis loops for all studied alloys, which were annealed in the presence of a transverse magnetic field HT = 140 kA/m at the temperature Ta = 525 °C/20 min.

From the hysteresis loops obtained for the samples heat-treated in the presence of the magnetic field, the distributions of a transverse anisotropy field as a second derivative of the returning branch from the saturation down to the remanent magnetization multiplied by (−*H*) [19] were determined:

(2) $$P\left(H_{k}\right)=-H\left(d^{2}m/dH^{2}\right)$$

where: *P*(*H_k_*)—distributions of a transverse anisotropy field; *H*—magnetic field and *m = B/B_s_*.

The value of the induced magnetic anisotropy *K_u_* was calculated using the Wolfhart-Stoner equation:

(3) $$H_{k}=\frac{2K_{u}}{\mu_{0}\cdot M_{s}}$$

where: *H_k_*—transverse anisotropy field; *K_u_*—induced magnetic anisotropy; *M_s_*—saturation magnetization.

The effect of the chemical composition of Fe_84.5__−x_Co_x_Nb_5_B_8.5_P_2_ nanocrystalline alloys, annealing temperature and the value of the transverse magnetic field on the induced magnetic anisotropy *K*_u_ are shown in Table 3. The value of saturation magnetization has been measured at H = 8000 A/m.

Based on the results presented in Table 3, it seems that a significant increase in the value of induced magnetic anisotropy from 30 J/m^3^ (for Co0) to 637 J/m^3^ (for Co20) is observed with the increase in Co content in the alloy. The later value applies to the material annealed at Ta = 525 °C/20 min in the presence of a transverse magnetic field of 140.3 kA/m. Induced transverse magnetic anisotropy occurs mainly in the crystalline phase and is caused by the increased ordering of atoms due to the formation of pairs of atoms with the orientation that ensures the most advantageous orientation of their magnetic moment with respect to the direction of the applied magnetic field. Two facts indicate this. Firstly, the anisotropy is induced during heating at a temperature much higher than the Curie temperature of an amorphous matrix (see Figure 11). The second reason is related to the existence of a close correlation, similar to that observed in Finemet modified by Co. The value of the magnetic anisotropy constant depends mainly on the content of the crystalline phase in the alloy [26]. In Pyroperm alloys with a higher cobalt content, we observe a clear and gradual decrease in the remanence of the cobalt-free values. Taking into account the reduction in coercivity, this leads to a reduction of the power losses in the core, even above 50% in the material with the highest cobalt content.

**Figure 13:**
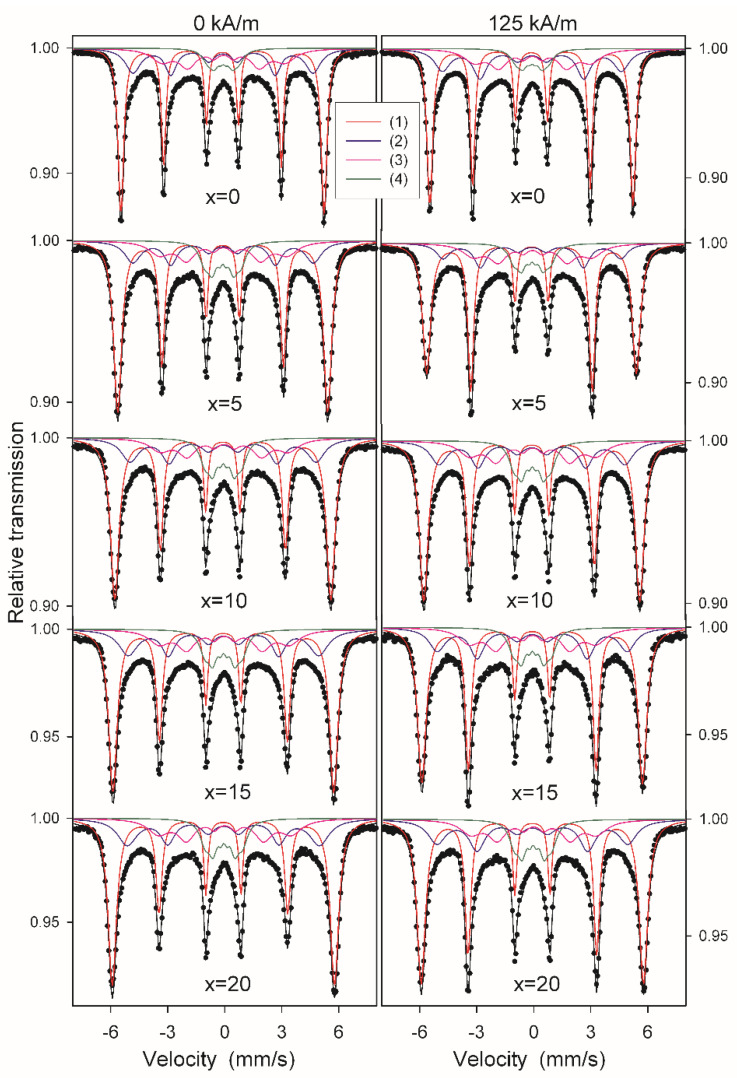
Mössbauer spectra of Fe_84.5__−x_Co_x_Nb_5_B_8.5_P_2_ alloys annealed at 525 °C for 20 min without (left column) and with (right column) the magnetic field of 125 kA/m.

**Figure 14:**
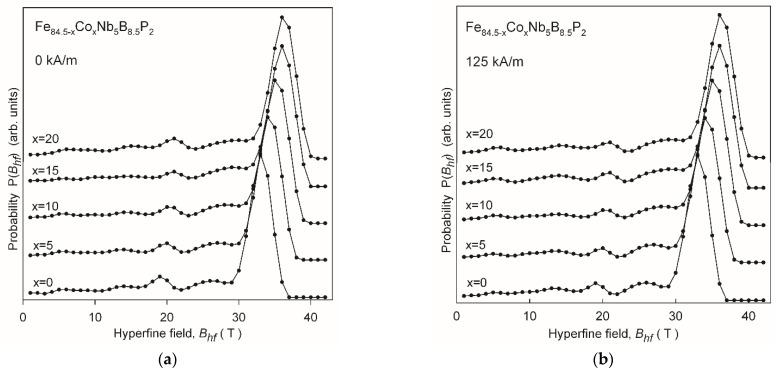
(**a**) Distributions of hyperfine fields determined from the Mössbauer spectra in Figure 13 (left column); (**b**) Distributions of hyperfine fields determined from the Mössbauer spectra in Figure 13 (right column).

The effect of the Co content on the phase composition and the influence of annealing in the external magnetic field on the magnetic spin orientation was investigated by the ^57^Fe Mössbauer spectroscopy method for Fe_84.5__−x_Co_x_Nb_5_B_8.5_P_2_ (x = 0–20%) alloys annealed at 525 °C for 20 min. Such annealing conditions ensured the completion of the primary nanocrystallization process for all studied alloys (see Figure 2) and were optimal for the magnetic properties and transverse induced anisotropy values. The Mössbauer spectra of the Pyroperm ribbons annealed without and in a magnetic field of 125 kA/m are shown in Figure 13. The spectra reveal a complex magnetic hyperfine structure. Therefore, they were initially fitted using a histogram method of a distribution of hyperfine field values. The calculated P(Bhf) distributions are plotted in Figure 14a,b. The main peak observed at the hyperfine field distributions P(Bhf) above 30T is related to the nanocrystalline phase. It shifts towards higher Bhf values, and its width significantly increases with increasing Co content in the alloy. This suggests the formation of a bcc Fe-based disordered solid solution with increasing Co concentration in the nanocrystalline phase. Minor peaks in the P(Bhf) distributions, which are observed in the range from about 4 to 22 T, originate from different atomic arrangements around Fe in the retained amorphous phase. For the peak at about 20 T, there is also a shift towards higher Bhf values with the increasing Co content in the alloy, which is less pronounced than in the case of the nanocrystalline peak. Thus the effect of Co concentration on the hyperfine field is more effective for the nanocrystalline phase than for the amorphous phase.

Afterwards, the Mössbauer spectra were fitted with discrete components, which allowed the qualitative and quantitative analysis of the phase composition (Figure 13). Due to the complex shape and on the basis of the P(Bhf) distributions, all spectra were fitted with four magnetically split components as follows:

(1) a sextet with Bhf1 ranging from 33.0 T to 36.3 T for x = 0 and x = 20, respectively,
(2) a sextet with Bhf2 ranging from 29.4 T to 31.4 T for x = 0 and x = 20, respectively,
(3) a sextet with Bhf3 ranging from 19.5 T to 21.7 T for x = 0 and x = 20, respectively,
(4) a sextet with Bhf4 ranging from 5.1 T to 6.1 T for x = 0 and x = 20, respectively.

The sextets (1) and (2) with hyperfine fields above 29 T originate from the nanocrystalline phase. The sextets (3) and (4) with much smaller Bhf and broad lines are assigned to inhomogeneous amorphous-like regions, which exhibit two different short-range atomic orders with higher and lower Fe content for (3) and (4) sextets, respectively. In the case of the crystalline components, the dominant sextet (1) is assigned to the bcc Fe phase for the Co-free alloy and to the bcc Fe(Co) solid solution for the Co5-Co20 alloys. The sextet (2) with a lower Bhf, much broader lines and a significantly smaller relative fraction compared to the sextet (1) can be attributed to Fe atoms located in crystalline interphase regions, the fraction of which is large enough for nanocrystalline materials to be detected by Mössbauer spectroscopy. The observed increase in the values of Bhf1 and Bhf2 along with the increase of Co content in the alloy shows the gradual substitution of Fe atoms with Co atoms in the nanocrystalline phase. Based on the experimental dependence of the hyperfine field on the Co concentration in the binary Fe-Co alloys [27], it was possible to estimate the chemical composition of the nanocrystalline phase. The approximate Co content in the bcc Fe(Co) phase is 6, 11, 16 and 20% for x = 5, 10, 15 and 20, respectively. Taking into account both sextets related to the crystal regions, it was found that the relative fraction of the nanocrystalline phase was similar for each alloy regardless of its chemical composition or the presence of an external magnetic field during annealing and was within a narrow range, from 73 to 74%. This indicates that the primary nanocrystallization was completed for all alloys. The effect of the presence of the external magnetic field during annealing is evidenced in the Mössbauer spectra by the change in the intensity of the second and fifth lines in sextets (1) and (2) related to the nanocrystalline phase. These lines have a much higher intensity for samples annealed in the presence of the external magnetic field than without such a field for all studied compositions (Figure 13). In general, intensities of the sextet lines are related to the angular dependence of the probability of the allowed nuclear spin transitions. The calculated ratio of the area of the second to third line of the discrete sextet (1) changes in a way that strongly suggests a change in spin orientation from random for standard annealing (0 kA/m) to more in-plane for the thermo-magnetic treatment (125 kA/m). The latter most likely corresponds to the direction of the applied transverse magnetic field. Thus, the Mössbauer spectra revealed that by applying the external magnetic field during annealing a preferential ordering of spins occurs in the nanocrystalline phase.

In order to check the applicability of the studied cores in power electronics in high-frequency and high-power conversion systems, their characteristic parameters, such as saturation induction Bs, remanence Br, coercivity Hc, magnetic permeability µ and core losses Ps, have been determined.

**Figure 15:**
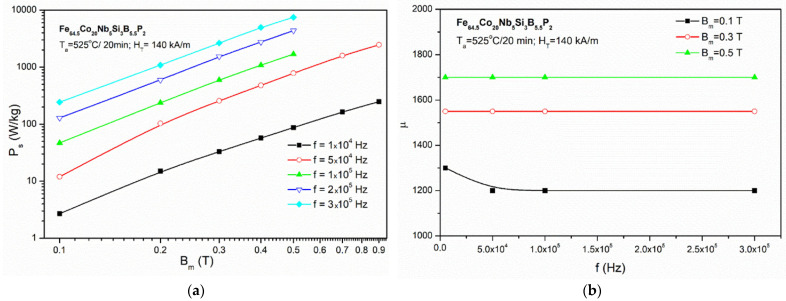
(**a**) Core losses (P_s_) for the Fe_64.5_Co_20_Nb_5_B_8.5_P_2_ core as a function of magnetization (B_m_) for 10–300 kHz; (**b**) magnetic permeability (µ) for the Fe_64.5_Co_20_Nb_5_B_8.5_P_2_ core as a function of frequency for B_m_ = 0.1–0.5 T.

The frequency dependence of Ps and µ up to 300 kHz at the field with magnetic induction up to 0.9 T obtained for the Fe_64.5_Co_20_Nb_5_B_8.5_P_2_ core is shown in Figure 15. It can be seen that the magnetic permeability of the Fe_64.5_Co_20_Nb_5_B_8.5_P_2_ alloy is almost constant at the level of 1700 (for Bm = 0.5 T) in the entire studied frequency range. This bring us to the conclusion that the Pyroperm-type alloy is suitable for application in high-power conversion systems.

**Figure 16:**
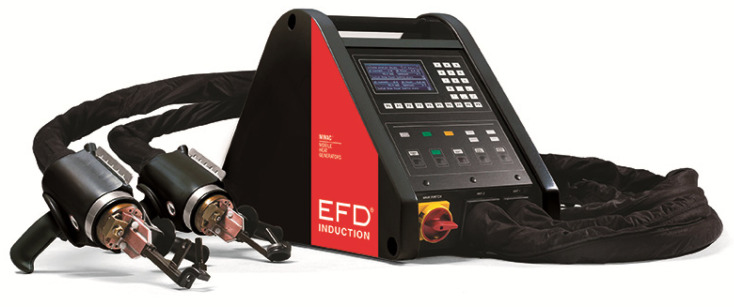
EFD Induction mobile induction heating equipment Miniac [28].

As a result of the research, two batches of nanocrystalline Fe_64.5_Co_20_Nb_5_B_8.5_P_2_ cores with a transverse induced magnetic anisotropy, weighing 1 kg and 0.5 kg, were prepared. The application of the cores in the Miniac, a mobile induction heating equipment (see Figure 16) (EFD Induction, Norway), made it possible to increase the induction heating power in a high-frequency range, required for use in a specific area related to market demand.

## 4. Conclusions

Detailed studies of Fe_84.5__−x_Co_x_Nb_5_B_8.5_P_2_ (x = 0–20 at.%) materials concerning the course of the crystallization process and the influence of the presence of a transverse magnetic field during annealing led to the determination of the optimal conditions for heat treatment (525 °C for 20 min in the presence of a 140 kA/m transverse magnetic field) in order to obtain the maximum value of induced magnetic anisotropy while maintaining a high value of saturation magnetization. Mössbauer spectroscopy studies confirmed the presence of Co in the nanocrystalline phase and the influence of the transverse magnetic field during annealing on the orientation of the magnetic spin, which has a direct impact on the magnitude of the induced magnetic anisotropy. The conducted high-frequency tests confirmed the stability of the parameters of Fe_64.5_Co_20_Nb_5_B_8.5_P_2_ cores as a function of frequency and the possibility of their application in power electronics in high-frequency and high-power conversion systems.

## Figures and Tables

**Table 1:** The influence of the Co content on the magnetic properties of Fe_84.5__−x_Co_x_Nb_5_B_8.5_P_2_ alloys annealed at 455 °C for 20 min, for which the hysteresis loops have been measured at 50 Hz, with a maximum applied field of 1500 A/m.

Sample	Bmax [T]	Br [T]	H_c_ [A/m]	P_s_(at Bmax) [W/kg]
Co0	1.62	0.444	31.1	1.4
Co5	1.66	0.556	31	1.2
Co10	1.7	0.622	28.4	1.4
Co15	1.68	0.646	31.9	1.4
Co20	1.64	0.461	44.2	1.72

**Table 2:** The influence of the annealing temperature Ta on the magnetic properties of the Fe_64.5_Co_20_Nb_5_B_8.5_P_2_ alloy, for which the hysteresis loops have been measured at 50 Hz, with a maximum applied field of 1500 A/m.

T_a_ [°C]	Bmax [T]	Br [T]	H_c_ [A/m]	P_s_ (at Bmax) [W/kg]
425	1.64	0.406	51.2	2.2
455	1.64	0.461	44.2	2
475	1.62	0.444	46.6	2.1
525	1.6	0.398	52.5	2.1
575	1.62	0.463	53.8	2.1
625	1.66	0.677	148	5.4
725	0.252	0.0969	598	2.7

**Table 3:** The influence of the transverse magnetic field H_T_ present during annealing on the anisotropy field H_k_ and induced transverse anisotropy K_u_ of the Fe_84.5__−x_Co_x_Nb_5_B_8.5_P_2_ (x = 0–20 at.%) cores.

Chemical Composition [at.%]	Annealing Temperature T_a_ [°C]	Transverse Magnetic Field H_T_ [kA/m]	Anisotropy Field H_k_ [A/m]	Saturation Magnetization M_s_ [T]	Induced Transverse Anisotropy K_u_ [J/m^3^]
Fe_64.5_Co_20_Nb_5_B_8.5_P_2_	525	140.3	750	1.7	637
		125.0	650		552
		109.8	647		549
		94.5	638		542
Fe_69.5_Co_15_Nb_5_B_8.5_P_2_	525	140.3	464	1.7	394
		125.0	429		364
		109.8	380		323
		94.5	367		311
Fe_74.5_Co_10_Nb_5_B_8.5_P_2_	525	140.3	238	1.7	202
		125.0	228		193
		109.8	226		192
		94.5	226		192
Fe_79.5_Co_5_Nb_5_B_8.5_P_2_	525	140.3	190	1.68	169
		125.0	180		151
		109.8	140		117
		94.5	130		109
Fe_84.5_Nb_5_B_8.5_P_2_	525	140.3	37	1.65	30
		125.0	35		28
		109.8	30		24
		94.5	28		23
